# Cancer Screening Patterns Among Current, Former, and Never Smokers in the United States, 2010-2015

**DOI:** 10.1001/jamanetworkopen.2019.3759

**Published:** 2019-05-17

**Authors:** Nina N. Sanford, David J. Sher, Santino Butler, Xiaohan Xu, Chul Ahn, Anthony V. D’Amico, Timothy Rebbeck, Ayal A. Aizer, Brandon A. Mahal

**Affiliations:** 1 Department of Radiation Oncology, University of Texas Southwestern Medical Center, Dallas; 2 Dana-Farber Cancer Institute/Brigham and Women’s Hospital, Harvard Medical School, Boston, Massachusetts; 3 Division of Biostatistics, Department of Clinical Sciences, University of Texas Southwestern Medical Center, Dallas; 4 Department of Statistical Science, Southern Methodist University, Dallas, Texas; 5 Harold Simmons Comprehensive Cancer Center, University of Texas Southwestern Medical Center, Dallas; 6 Department of Radiation Oncology, Brigham and Women’s Hospital, Boston, Massachusetts; 7 Department of Medical Oncology, Dana-Farber Cancer Institute, Boston, Massachusetts; 8 McGraw/Patterson Center for Population Sciences, Dana-Farber Cancer Institute, Boston, Massachusetts

## Abstract

**Importance:**

National guidelines recommend screening for several cancer types, yet screening rates remain below target. To date, cancer screening patterns by smoking status, a major cancer risk factor, are unknown.

**Objective:**

To assess cancer screening patterns among individuals who never smoked (never smokers), formerly smoked (former smokers), and currently smoke (current smokers) in the United States.

**Design, Setting, and Participants:**

This cross-sectional study assessed data from the National Health Interview Survey years 2010, 2013, and 2015. Adult participants (aged ≥18 years) who had never reported a cancer diagnosis were included in the analysis. Data were analyzed from August 1, 2018, through February 1, 2019.

**Exposures:**

Receipt of cancer screening, including colonoscopy, mammography, prostate-specific antigen testing, and Papanicolaou test per the US Preventive Services Task Force guidelines.

**Main Outcomes and Measures:**

Multivariable logistic regression defined adjusted odds ratios (AORs) and 95% CIs for undergoing cancer screening by smoking status. Among participants who received a specific screening test, AORs and 95% CIs of receiving the test within guideline-recommended intervals were also assessed.

**Results:**

Among 83 176 participants (45 851 [55.1%] women; mean [SD] age, 47 [18] years), 51 014 (61.3%) were never smokers; 17 235 (20.7%), former smokers; and 14 927 (17.9%), current smokers. Compared with never smokers, current smokers were less likely to ever have received a colonoscopy (43.8% vs 57.7%; AOR, 0.74; 95% CI, 0.68-0.82; *P* < .001), mammogram (88.8% vs 93.3%; AOR, 0.70; 95% CI, 0.57-0.87; *P* = .001), or prostate-specific antigen test (46.1% vs 60.8%; AOR, 0.76; 95% CI, 0.64-0.90; *P* = .001). Among those who had ever received a specific screening test, current smokers were less likely to have undergone colonoscopy (92.1% vs 95.1%; AOR, 0.75; 95% CI, 0.59-0.96; *P* = .02), mammography (62.4% vs 79.4%; AOR, 0.52; 95% CI, 0.45-0.60; *P* < .001), or Papanicolaou test (80.9% vs 90.8%; AOR, 0.61; 95% CI, 0.56-0.67; *P* < .001) within the recommended time frame compared with never smokers. Former smokers were more likely than never smokers to undergo any of the screening studies evaluated, with the exception of prostate-specific antigen screening (colonoscopy, 65.2% vs 57.7%; AOR, 1.20; 95% CI, 1.12-1.30; *P* < .001; mammography, 95.7% vs 93.3%; AOR, 1.35; 95% CI, 1.07-1.70; *P* = .01; Papanicolaou test, 97.6% vs 91.4%; AOR, 2.51; 95% CI, 1.93-3.26; *P* < .001).

**Conclusions and Relevance:**

This study found that current smokers appeared to be less likely to receive guideline-concordant screening studies for breast, prostate, and colorectal cancer compared with never smokers. Further research is needed to identify barriers to screening among current smokers with the goal of increasing acceptance and uptake of cancer screening among this population at high risk of cancer.

## Key Points

**Question:**

Are individuals who currently smoke (current smokers) less likely to undergo age-appropriate cancer screening than never and former smokers?

**Findings:**

In this nationally representative, cross-sectional study of 83 176 participants, current smokers were less likely to adhere to US Preventive Services Task Force screening guidelines for colonoscopy, mammography, or prostate-specific antigen testing compared with never smokers.

**Meaning:**

This study found that disparities in cancer screening occurred among current smokers, which may prompt initiatives to increase uptake of cancer screening in this population at high risk for cancer.

## Teaser

This cross-sectional study using National Health Interview Survey data assesses patterns of cancer screening among never, former, and current adult smokers in the United States in 2010-2015.

## Introduction

The widespread use of cancer screening has reduced mortality due to several cancer types, including cervical and colorectal, during the past several decades.^1,2,3^ Healthy People 2020 was launched in 2010 with objectives to promote evidence-based cancer screening per US Preventive Services Task Force (USPSTF) recommendations during the upcoming decade.^4^ A 2015 publication showed that most US populations did not meet Healthy People 2020 targets for cancer screening and that significant disparities in screening uptake existed based on race/ethnicity, income, and educational attainment.^5^

Recent reports, however, have not assessed the association between smoking status and adherence to USPSTF cancer screening guidelines. Since the USPSTF gave lung cancer screening a grade B recommendation in 2013, rates of low-dose computed tomography of the chest have increased, albeit not without concerns regarding slow uptake in eligible individuals and overuse in others.^6^ In addition to lung cancer, smoking has been linked to cancer risk at additional sites, including the colorectal tract and cervix, with emerging evidence on other cancer types.^7,8^ Thus, although smoking cessation remains the most critical cancer prevention behavior, individuals who smoke may potentially derive the greatest benefit from improved cancer screening to detect occult disease at an earlier stage. As such, an assessment of cancer screening patterns among smokers is needed. We used a comprehensive nationwide database to examine cancer screening behaviors by smoking status.

## Methods

### Data Source

The National Health Interview Survey (NHIS) is a cross-sectional household survey of noninstitutionalized civilian adults living in the United States that assesses a wide range of health status and utilization measures.^9^ The NHIS was first administered in 1957 and uses a multistage probability design to ensure broad geographic representation. Sample weights are provided for each individual, permitting inference on national prevalence. Beginning in 1987, the NHIS has periodically administered a cancer control supplement focusing on issues pertaining to knowledge, attitudes, and practices of cancer-related health behaviors, screening, and risk assessment. Harmonized data were obtained through the Integrated Health Interview Series.^10^ Data for each study were collected in 2010, 2013, and 2015. The NHIS is overseen by the National Center for Health Statistics research ethics review board. Survey participants provided verbal informed consent. The institutional review board of University of Texas Southwestern Medical Center, Dallas, deemed this study to be exempt from ethics review given the use of public deidentified data. Data and analyses are presented in accordance with the Strengthening the Reporting of Observational Studies in Epidemiology (STROBE) reporting guideline for cross-sectional studies.^11^

### Population

Our study population included adults (aged ≥18 years) surveyed in 2010, 2013, and 2015, the only years since 2010 for which cancer screening data were available. Participants who reported ever having a diagnosis of cancer were excluded (7738 out of 90 914 total population). Additional demographic variables, including smoking status, age, sex, race, ethnicity, income, insurance status, and educational attainment, were queried. For smoking status, participants were asked “Have you smoked at least 100 cigarettes during your entire life?” Those who responded no were categorized as individuals who never smoked (never smokers). Those who responded yes were further asked “Do you NOW smoke cigarettes every day, some days, or not at all?” and categorized as individuals who currently smoke every day, who currently smoke some days, or who formerly smoked (former smokers), respectively. For the purpose of our analysis, individuals who reported currently smoking every day and some days were combined into the classification current smokers. Participants who were current smokers were also asked whether they had attempted quitting for more than 1 day during the past year. In addition, a 9-item comorbidity index was created based on self-reported chronic conditions, including hypertension, coronary heart disease, stroke, chronic obstructive pulmonary disease, asthma, diabetes, arthritis, hepatitis, and weak or failing kidneys.^12,13,14^ Participants were categorized as having 0, 1, or 2 or more chronic conditions.

### Cancer Screening Measures

We examined the following 4 cancer screening measures: colonoscopy, testing for prostate-specific antigen (PSA) level, mammography, and Papanicolaou test. We did not evaluate use of screening computed tomography of the chest for smokers because an analysis would have been confounded by indication. Participants were first asked whether they had ever undergone the specific screening measure. Those who answered yes were then asked when their most recent screening test occurred.

The most recent USPSTF guidelines were used for age and frequency of colonoscopy, Papanicolaou test, PSA testing, and mammography.^15,16,17,18,19^ The USPSTF grades include A and B for recommends this service, C for recommends selectively offering or providing this service to individual patients based on professional judgment and patient preferences, and D for recommends against this service.^20^ The guidelines included colonoscopy within 10 years for adults aged 50 to 75 years (grade A), Papanicolaou test within 3 years for women aged 21 to 65 years without prior hysterectomy (grade A), PSA testing for men aged 55 to 69 years (grade D, upgraded to C in 2017), and biennial mammography for women aged 50 to 74 years (grade B). Notably, prostate cancer screening guidelines per USPSTF have changed significantly during the last decade. In 2008, screening was given a grade I recommendation (insufficient evidence to assess benefits of balance and harms) for men younger than 75 years and grade D for men 75 years and older.^21^ In 2012, the recommendation was changed to grade D for all men,^22^ and more recently in 2017, men aged 55 to 69 years were given a grade C recommendation, whereas the recommendation for those 70 years and older remained grade D.^15^

### Statistical Analysis

#### Baseline Characteristics

Baseline characteristics of the cohort stratified by smoking status were reported. The Kruskal-Wallis and χ^2^ tests compared distributions of continuous and categorical covariates, respectively.

#### Cancer Screening by Smoking Status

The primary independent variable of interest was smoking status, and the primary end point of interest was receipt of cancer screening. For each of the screening tests (colonoscopy, PSA test, mammography, and Papanicolaou test), multivariable logistic regression defined adjusted odds ratio (AOR) and associated 95% CIs of cancer screening by smoking status. Additional variables adjusted for and included in the models were age, sex, race, ethnicity, annual family income, insurance status, highest educational level attained, and comorbidity score. Weighted prevalence of cancer screening by smoking status was also estimated.

For participants who reported ever undergoing a specific cancer screening test, multivariable logistic regression defined the AOR and 95% CI of receiving screening within the recommended time frame (adjusted for the variables listed above) as defined by the USPSTF guidelines above; weighted prevalence of this end point was also assessed. As a secondary end point, the aforementioned analyses were repeated among current smokers who attempted to quit smoking during the past 12 months vs not as the independent variable of interest. Outcomes were measured at the time of survey in this cross-sectional study with no follow-up time. All analyses were restricted to the specific populations for whom testing was recommended per USPSTF guidelines (eg, analyses for mammography included only women aged 50-74 years).

#### Prostate Cancer Screening by Smoking Status Stratified by Year

Given changing prostate cancer screening recommendations, multivariable logistic regression defined the AOR and 95% CI of ever receiving PSA testing for men aged 50 to 70 years in 2010, 2013, and 2015. Models were adjusted for demographic and socioeconomic status variables described above. This age range was chosen because it comprehensively includes age cutoffs in previous USPSTF guidelines along with those issued by other medical societies. These organizations include the American Cancer Society, which recommends discussion of PSA testing starting at age 50 years for men at average risk^23^; the American Urological Society, which encourages men aged 55 to 69 years to undergo shared decision making regarding PSA testing^24^; and the Mayo Clinic, which recommends screening for men aged 50 to 70 years.^25^

Sample weighting stratified by year was used for all analyses to produce nationally representative estimates. Statistical testing was 2-sided, with α = .05. All AORs and accompanying 95% CIs were generated using multivariable logistic regressions. Analyses were performed with Stata/SE, version 15.1 (StataCorp), or R, version 3.0.2 (R Project for Statistical Computing).

## Results

### Baseline Characteristics

Among 83 176 participants (45 851 [55.1%] women; mean [SD] age, 47 [18] years), 51 014 (61.3%) were never smokers; 17 235 (20.7%), former smokers; and 14 927 (17.9%), current smokers (Table 1). Former smokers were older (mean [SD] age, 55 [17] years; *P* < .001) than never smokers (mean [SD] age, 45 [18] years) or current smokers (mean [SD] age, 44 [15] years. Compared with former and never smokers, current smokers tended to have lower annual family income (<$35 000/y, 34.9% and 36.8%, respectively, vs 53.1%), were more likely to be uninsured (uninsured, 10.9% and 15.5%, respectively, vs 26.0%), and achieved lower educational levels (≥5 years of college, 9.4% and 12.4%, respectively, vs 3.4%). (Table 1).

**Table 1.** Distribution of Baseline Patient Characteristics by Smoking Status Among US Participants Aged 18 and Older Not Reporting a History of Cancer Surveyed in 2010, 2013, and 2015^a^

Smoking Status	Total (N = 83 176)	Smoking Status		
		Never (n = 51 014)	Former (n = 17 235)	Current (n = 14 927)
Sex				
Male	37 325 (44.9)	20 465 (40.1)	9087 (52.7)	7773 (52.1)
Female	45 851 (55.1)	30 549 (59.9)	8148 (47.3)	7154 (47.9)
Race^b^				
White	62 620 (75.3)	37 015 (72.6)	14 244 (82.6)	11 361 (76.1)
Black	13 456 (16.2)	8827 (17.3)	1975 (11.5)	2654 (17.8)
Native Alaskan or American	1032 (1.2)	562 (1.1)	212 (1.2)	258 (1.7)
Chinese	5532 (6.7)	4266 (8.4)	709 (4.1)	557 (3.7)
Unknown/multiple	536 (0.6)	344 (0.7)	95 (0.6)	97 (0.6)
Ethnicity^b^				
Non-Spanish/non-Hispanic/ non-Latino	67 722 (81.4)	39 678 (77.8)	15 044 (87.3)	13 000 (87.1)
Spanish, Hispanic, or Latino	15 454 (18.6)	11 336 (22.2)	2191 (12.7)	1927 (12.9)
Age, mean (SD), y	47 (18)	45 (18)	55 (17)	44 (15)
Annual income, US$				
0-34 999	32 697 (39.3)	18 757 (36.8)	6020 (34.9)	7920 (53.1)
35 000-74 999	23 842 (28.7)	14 474 (28.4)	5247 (30.4)	4121 (27.6)
75 000-99 999	7963 (9.6)	5081 (10.0)	1819 (10.6)	1063 (7.1)
≥100 000	13 420 (16.1)	9298 (18.2)	2976 (17.3)	1146 (7.7)
Not ascertained/unknown	5254 (6.3)	3404 (6.7)	1173 (6.8)	677 (4.5)
Insurance				
Insured	69 189 (83.2)	42 884 (84.1)	15 310 (88.8)	10 995 (73.7)
Not insured	13 677 (16.4)	7907 (15.5)	1883 (10.9)	3887 (26.0)
Unknown	310 (0.4)	223 (0.4)	42 (0.2)	45 (0.3)
Highest educational level attained				
None or grades K-4	1313 (1.6)	931 (1.8)	212 (1.2)	170 (1.1)
Grades 5-12	32 538 (39.1)	17 806 (34.9)	6854 (39.8)	7878 (52.8)
1-4 y college	40 523 (48.7)	25 717 (50.4)	8489 (49.3)	6317 (42.3)
≥5 y college	8466 (10.2)	6345 (12.4)	1619 (9.4)	502 (3.4)
Unknown	336 (0.4)	215 (0.4)	61 (0.4)	60 (0.4)
Comorbidity				
0	41 811 (50.3)	28 429 (55.7)	6148 (35.7)	7234 (48.5)
1	22 124 (26.6)	12 900 (25.3)	5036 (29.2)	4188 (28.1)
≥2	19 241 (23.1)	9685 (19.0)	6051 (35.1)	3505 (23.5)

^a^ Data are obtained from the National Health Interview Survey years 2010, 2013, and 2015. Unless otherwise indicated, data are expressed as number (percentage) of participants. Percentages have been rounded and may not total 100. *P* < .001 when comparing across smoking status for all baseline characteristics.

^b^ Race and ethnicity were self-reported as captured by the National Health Interview Survey. Participants were asked whether they identified with 1 or more of the following racial groups: white, black/African American, Native Alaskan or American, Chinese, Filipino, Asian Indian, or other Asian. Those reporting Chinese, Filipino, Asian Indian, or other Asian race were grouped as Asian. Participants with multiple races or primary race not releasable were grouped as unknown/multiple. Participants were also asked whether they identified with 1 or more of the following ethnicities: non-Hispanic/non-Spanish origin, Mexican, Mexican American, Puerto Rican, Cuban/Cuban American, Dominican (Dominican Republic), Central or South American, other Latin American (type not specific), other Spanish, or multiple Hispanic. Those reporting any of the ethnicities with the exception of non-Hispanic/non-Spanish origin were categorized as Spanish-Hispanic-Latino.

### Cancer Screening by Smoking Status

**Figure 1.**
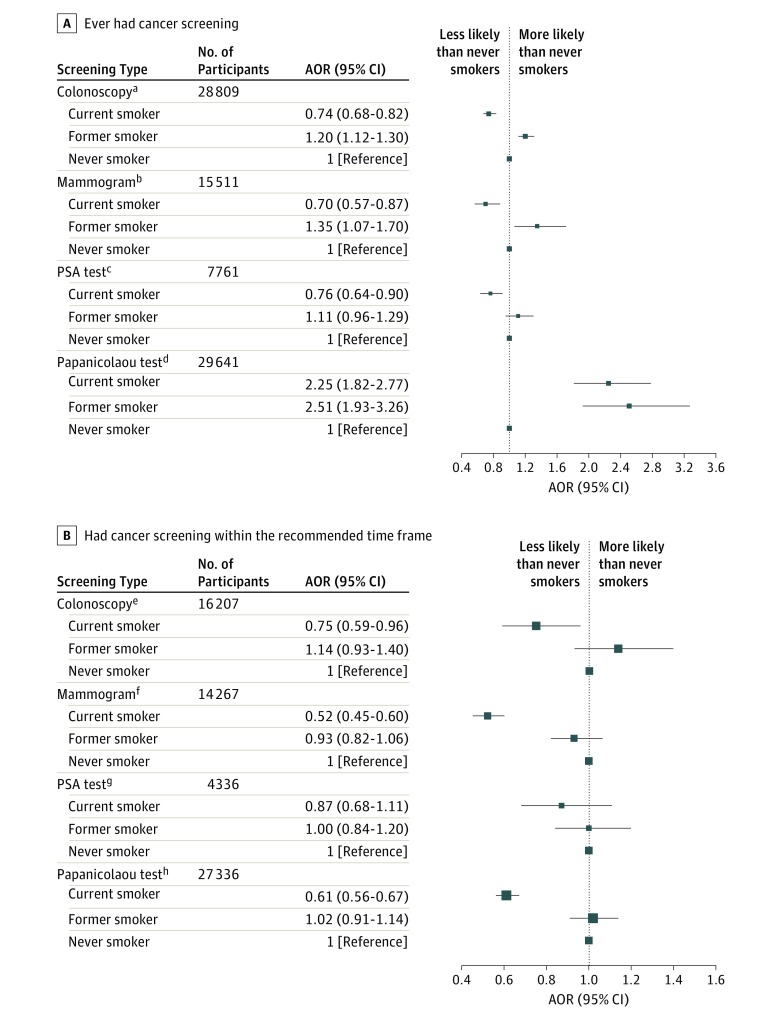
Forest Plot Depicting Adjusted Odds Ratios (AORs) of Cancer Screening for Former and Current Smokers Never smokers served as the reference group. PSA indicates prostate-specific antigen. The size of each data point is inversely related to the 95% CI size. ^a^Includes adults aged 50 to 75 years. ^b^Includes women aged 50 to 74 years. ^c^Includes men aged 55 to 69 years. ^d^Includes women aged 21 to 65 years without prior hysterectomy. ^e^Indicates within 10 years. ^f^Indicates within 2 years. ^g^Indicates within 1 year. ^h^Indicates within 3 years.

**Figure 2.**
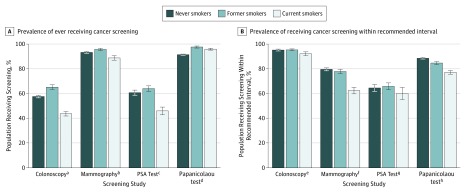
Weighted Prevalence of Receiving Cancer Screening Error bars indicate 95% CIs; PSA, prostate-specific antigen. ^a^Includes adults aged 50 to 75 years. ^b^Includes women aged 50 to 74 years. ^c^Includes men aged 55 to 69 years. ^d^Includes women aged 21 to 65 years without prior hysterectomy. ^e^Indicates within 10 years. ^f^Indicates within 2 years. ^g^Indicates within 1 year. ^h^Indicates within 3 years.

Compared with never smokers, current smokers meeting USPSTF criteria for screening were less likely to ever have received a colonoscopy (43.8% vs 57.7%; AOR, 0.74; 95% CI, 0.68-0.82; *P* < .001), mammogram (88.8% vs 93.3%; AOR, 0.70; 95% CI, 0.57-0.87; *P* = .001), or PSA test (46.1% vs 60.8%; AOR, 0.76; 95% CI, 0.64-0.90; *P* = .001) (Figure 1A and Figure 2A). In contrast, current smokers were more likely to have ever undergone a Papanicolaou test (95.9% vs 91.4%; AOR, 2.25; 95% CI, 1.82-2.77; *P* < .001) compared with never smokers. Former smokers were more likely than never smokers to undergo any of the screening studies evaluated, with the exception of a PSA test (colonoscopy, 65.2% vs 57.7% [AOR, 1.20; 95% CI, 1.12-1.30; *P* < .001]; mammography, 95.7% vs 93.3% [AOR, 1.35; 95% CI, 1.07-1.70; *P* = .01]; Papanicolaou test, 97.6% vs 91.4% [AOR, 2.51; 95% CI, 1.93-3.26; *P* < .001]) (Figure 1A and Figure 2A).

Among those who had ever received a specific screening test, current smokers were less likely to have undergone colonoscopy (92.1% vs 95.1%; AOR, 0.75; 95% CI, 0.59-0.96; *P* = .02), mammography (62.4% vs 79.4%; AOR, 0.52; 95% CI, 0.45-0.60; *P* < .001), or Papanicolaou test (80.9% vs 90.8%; AOR, 0.61; 95% CI, 0.56-0.67; *P* < .001) within the recommended time frame compared with never smokers (Figure 1B). For example, 62.4% of current female smokers aged 50 to 74 years had undergone mammography in the previous 2 years, compared with 79.5% of never smokers and 77.8% of former smokers. The proportions of participants who had ever undergone a screening study and who had received the screening study within the recommended interval are shown in Figure 2. Notably, the absolute differences in screening rates between never and current smokers for PSA screening and colonoscopy approached 15%, whereas that for mammography was more modest at 4.5%.

Among 14 927 current smokers, 7161 (48.0%) reported attempting to quit smoking for at least 1 day during the past 12 months. Those who had attempted smoking cessation were more likely to have ever undergone colonoscopy (47.6% vs 41%; AOR, 1.25; 95% CI, 1.07-1.46; *P* = .005) and mammography (92.6% vs 86.0%; AOR, 2.02; 95% CI, 1.38-2.94; *P* < .001) compared with those who had not and also more likely to undergo mammography (66.3% vs 59.1%; AOR, 1.30; 95% CI, 1.02-1.66; *P* = .03) and Papanicolaou test (83.9% vs 77.4%; AOR, 1.30; 95% CI, 1.06-1.58; *P* = .01) within the recommended time frame (Table 2).

**Table 2.** Odds of Ever Undergoing Cancer Screening and Undergoing Screening Within the Recommended Time Interval Among Current Smokers Who Attempted to Quit in the Past Year vs Those Who Did Not^a^

Recommended Screen	Attempted to Quit in Past Year (n = 14 927)	
	AOR (95% CI)^b^	*P* Value
Colonoscopy in adults aged 50-75 y (n = 5246)		
Ever	1.25 (1.07-1.46)	.005
Within 10 y	1.22 (0.82-1.84)	.33
Mammography in women aged 50-74 y (n = 2483)		
Ever	2.02 (1.38-2.94)	<.001
Within 2 y	1.30 (1.02-1.66)	.03
PSA test in men aged 55-69 y (n = 1609)		
Ever	1.16 (0.87-1.54)	.32
Within 1 y	1.09 (0.72-1.65)	.68
Papanicolaou test in women aged 21-65 y (n = 5070)^c^		
Ever	1.14 (0.79-1.65)	.48
Within 3 y	1.30 (1.06-1.58)	.01

Abbreviations: AOR, adjusted odds ratio; PSA, prostate-specific antigen.

^a^ Includes participants not reporting a cancer diagnosis in the National Health Interview Survey years 2010, 2013, and 2015. Those not attempting to quit constituted the reference category for the primary comparison.

^b^ All AORs are adjusted for the following sociodemographic variables: age (continuous), sex (male [reference] and female), race (white [reference], black, Alaskan, Asian, and other), ethnicity (non-Hispanic [reference] and Hispanic), annual family income (0-$34 999 [reference], $35 000-$74 999, $75 000-$99 999, ≥$100 000, and unknown), insurance status (no coverage [reference], has coverage, and coverage status unknown), highest educational level attained (none/kindergarten only [reference], grades 1-8, grades 9-12, 1-4 years of college, ≥5 years of college, and unknown) and comorbidity score (0 [reference], 1, and ≥2).

^c^ Excludes women with prior hysterectomy.

### Prostate Cancer Screening by Smoking Status Stratified by Year

No difference in PSA screening between current and never smokers occurred in 2010 (42.6% vs 50.7%; AOR, 0.85; 95% CI, 0.67-1.10; *P* = .21) (Table 3). In contrast, the odds of ever undergoing PSA testing were significantly lower in current smokers than never smokers in 2013 (38.3% vs 51.7%; AOR, 0.77; 95% CI, 0.61-0.98; *P* = .03) and 2015 (39.9% vs 55.1%; AOR, 0.72; 95% CI, 0.56-0.93; *P* = .01) (with grade D USPSTF recommendations against PSA testing) (Table 3).

**Table 3.** Odds of Ever Undergoing PSA Testing Stratified by Year^a^

Characteristic	Survey Year					
	2010 (n = 2954)		2013 (n = 4342)		2015 (n = 4219)	
	AOR (95% CI)	*P* Value	AOR (95% CI)	*P* Value	AOR (95% CI)	*P* Value
Smoking status						
Never	1 [Reference]		1 [Reference]		1 [Reference]	
Former	1.26 (1.00-1.57)	.05	0.99 (0.81-1.20)	.90	1.20 (0.97-1.48)	.10
Current	0.85 (0.67-1.10)	.21	0.77 (0.61-0.98)	.03	0.72 (0.56-0.93)	.01
Age	1.10 (1.08-1.12)	<.001	1.09 (1.07-1.11)	<.001	1.07 (1.05-1.09)	<.001
Race^b^						
White	1 [Reference]		1 [Reference]		1 [Reference]	
Black	1.14 (0.88-1.49)	.32	1.12 (0.87-1.43)	.37	1.10 (0.85-1.44)	.46
Native Alaskan or American	0.52 (0.15-1.85)	.31	0.86 (0.40-1.85)	.70	1.17 (0.55-2.49)	.68
Chinese	0.52 (0.34-0.79)	.002	0.49 (0.33-0.72)	<.001	0.28 (0.19-0.42)	<.001
Unknown	0.18 (0.02-1.92)	.16	0.52 (0.10-2.64)	.43	0.99 (0.26-3.82)	.99
Ethnicity^b^						
Non-Spanish/non-Hispanic/non-Latino	1 [Reference]		1 [Reference]		1 [Reference]	
Spanish, Hispanic, or Latino	0.70 (0.52-0.94)	.02	0.79 (0.60-1.04)	.09	0.72 (0.55-0.96)	.02
Income, US$						
0-34 999	1 [Reference]		1 [Reference]		1 [Reference]	
35 000-74 999	1.31 (1.03-1.67)	.03	1.58 (1.26-1.99)	<.001	1.36 (1.07-1.73)	.01
75 000-99 999	1.49 (1.05-2.11)	.02	1.38 (1.00-1.91)	.5	1.83 (1.32-2.52)	<.001
≥100 000	1.81 (1.32-2.47)	<.001	2.36 (1.81-3.09)	<.001	2.22 (1.68-2.92)	<.001
Unknown	1.92 (1.19-3.09)	.008	2.25 (1.56-3.26)	<.001	1.56 (1.10-2.21)	.01
Insurance						
Insured	1 [Reference]		1 [Reference]		1 [Reference]	
Not insured	0.54 (0.39-0.74)	<.001	0.61 (0.46-0.82)	.001	0.58 (0.41-0.82)	.002
Highest educational level attained						
None or grades K-4	1 [Reference]		1 [Reference]		1 [Reference]	
Grades 5-12	1.37 (0.62-3.03)	.44	0.60 (0.33-1.11)	.11	0.77 (0.36-1.64)	.49
1-4 y college	2.43 (1.09-5.43)	.03	0.92 (0.49-1.71)	.78	1.24 (0.58-2.67)	.58
≥5 y college	3.69 (1.57-8.65)	.003	1.44 (0.74-2.82)	.29	1.96 (0.87-4.40)	.10
Unknown	0.71 (0.16-3.16)	.66	0.23 (0.05-1.08)	.06	0.36 (0.05-2.52)	.30
Comorbidity, No. (%)						
0	1 [Reference]		1 [Reference]		1 [Reference]	
1	1.41 (1.12-1.78)	.004	2.22 (1.79-2.75)	<.001	1.55 (1.23-1.95)	<.001
≥2	1.74 (1.38-2.20)	<.001	2.28 (1.84-2.82)	<.001	1.78 (1.41-2.24)	<.001

Abbreviations: AOR, adjusted odds ratio; PSA, prostate-specific antigen.

^a^ Includes 11 425 men aged 50 to 70 years in the National Health Interview Survey years 2010, 2013 and 2015.

^b^ Race and ethnicity were self-reported as captured by the National Health Interview Survey. Participants were asked whether they identified with 1 or more of the following racial groups: white, black/African American, Native Alaskan or American, Chinese, Filipino, Asian Indian, or other Asian. Those reporting Chinese, Filipino, Asian Indian, or other Asian race were grouped as Asian. Participants with multiple races or primary race not releasable were grouped as unknown/multiple. Participants were also asked whether they identified with 1 or more of the following ethnicities: non-Hispanic/non-Spanish origin, Mexican, Mexican American, Puerto Rican, Cuban/Cuban American, Dominican (republic), Central or South American, other Latin American (type not specific), other Spanish, or multiple Hispanic. Those reporting any of the ethnicities with the exception of non-Hispanic/non-Spanish origin were categorized as Spanish-Hispanic-Latino.

## Discussion

In this large, contemporary, national survey, current smokers were less likely to receive guideline-concordant screening for breast, prostate, and colorectal cancer than were never smokers after correcting for relevant demographic and socioeconomic factors. In contrast, former smokers were more likely than never smokers to receive breast, prostate, and colorectal cancer screening at age-appropriate intervals. Furthermore, among current smokers, those who attempted to quit were more likely to have been screened for breast and colorectal cancer than were smokers who did not attempt to quit.

The lower rate of cancer screening in current smokers despite their increased cancer risk is a concerning paradox. As such, our study has several major public health implications. First, by foregoing age-appropriate cancer screening, smokers are at risk for presenting with later-stage cancers. Smoking is associated with a wide range of adverse outcomes after a cancer diagnosis,^26,27^ and a later stage of diagnosis could therefore further exacerbate cancer outcomes among this population. A previously published report including 954 029 individuals^28^ found that current smokers had 17% excess mortality compared with those who never smoked. In that study, current smoking was associated with increased risk of death due to cancers of the prostate and breast, disease sites where a causal link between smoking and cancer development has not yet been firmly established. Our study raises the hypothesis that lower rates of screening among current smokers could lead to more advanced presentation and contribute to their worse outcomes. Second, our study identifies a high-risk population that appears to be receiving suboptimal cancer screening that would benefit from measures to promote screening and to meet Healthy People 2020 targets. Although further research is needed to elucidate specific barriers to screening for individuals who smoke, policy and interventions may be warranted to support appropriate cancer screening among this population. Importantly, increased efforts directed toward cancer screening in individuals who smoke may also provide an impetus and greater support for cessation of tobacco use.

The reasons for lower cancer screening rates among smokers are likely multifactorial. Prior studies examining beliefs about cancer and health-seeking behaviors^29^ found that current smokers were more likely to hold pessimistic and avoidant beliefs about cancer. In particular, current smokers were more likely to view cancer as a death sentence and to believe that a diagnosis of cancer would preclude continuation of normal activities.^30^ These observations suggest that smokers’ awareness regarding the negative effects of tobacco use may actually discourage them from undergoing cancer screening. Prior research has also shown an association between heavy smoking and other adverse health choices, such as consuming junk food, decreased exercise, and greater alcohol use.^31,32^ This clustering of unhealthy practices raises the hypothesis that health behavior choices, including the decision to undergo cancer screening, may be a reflection of personal biases regarding health status. Former smokers and the subset of current smokers who have recently attempted to quit may feel especially motivated to take initiatives to optimize their long-term health. Ultimately, further research is needed to better understand the multifactorial causes of the observed cancer screening disparities among smokers.

Our finding that current and former smokers were more likely to undergo Papanicolaou tests was not surprising, given the multiple indications for Papanicolaou tests outside of cancer screening. We hypothesize 3 potential explanations for this association. First, Papanicolaou tests can be performed efficiently in office by a primary care physician or a gynecologist. In contrast, colonoscopies or mammograms require an additional visit, preprocedural preparation, and/or an appointment with a specialist. Notably however, PSA screening can also be easily performed in office; thus, this rationale would not explain the increased uptake of Papanicolaou tests compared with PSA testing among smokers. Second, prior research has shown an association between smoking and sexually transmitted infections.^33,34^ Seeking care for sexually transmitted infections may present smokers an opportunity to undergo Papanicolaou tests. Third, studies have suggested an association between smoking and sexual activity.^35^ Young women who are interested in becoming sexually active may approach their physicians regarding contraception, which historically has been accompanied by a pelvic examination, including a Papanicolaou test. Accordingly, the rate of ever having a Papanicolaou test would be higher within this group, but the rate of undergoing Papanicolaou tests within the recommended time frame would be lower, which was observed in our study.

The emerging disparity in PSA testing warrants further discussion. Notably, no difference in odds of undergoing screening was found in 2010, when the USPSTF did not issue guidelines against PSA testing. However, in 2013 and 2015, after PSA testing received a grade D recommendation, current smokers were less likely than never smokers to receive the test. This finding suggests that although guidelines are created to standardize practices, they may allow for existing disparities to become apparent. Since the USPSTF upgraded their recommendations from grade D to C in 2017, experts foresee an increased uptake in PSA testing overall. We await the next NHIS data release to ascertain how this change may have differentially affected the smoking population.

### Strengths and Limitations

Most prior research on factors associated with cancer screening have shown a negative association between adherence to screening recommendations and smoking status. However, these studies were conducted before the publication of current screening guidelines, have been limited to women, were based on foreign participants or individuals residing in a specific US state, or only included select cancer types.^36,37,38,39,40,41,42,43^ These studies are therefore not representative of current nationwide cancer screening trends among the smoking population.

Notwithstanding, our study has notable limitations. First, all data were self-reported, including smoking status and interval since cancer screening. However, research has shown high concordance between patient- and physician-reported smoking behavior.^44,45^ Published rates on agreement for report of cancer screening appear to vary, with most studies showing higher self-reported rates than what were supported by the medical record.^46,47^ Second, the survey did not distinguish whether a test was administered for screening or diagnostic purposes. By excluding participants with a cancer diagnosis from our analyses, however, we would expect most tests to have been performed for screening. Third, the timing of follow-up screening can depend on results from the prior examination. Because information was not available regarding results of any prior screening study, we chose the recommended interval for individuals with normal findings, acknowledging that this interval may fall outside the time frame for those with abnormal examination findings. Fourth, our study focused on the primary screening methods for colorectal, prostate, breast, and cervical cancer and did not analyze the association between smoking status and uptake of other test types used less frequently, such as fecal occult blood testing for colorectal cancer. Fifth, given that PSA testing received a grade D recommendation in 2012, the decreased uptake of PSA testing in 2013 and 2015 among smokers does not technically represent lower guideline compliance in this population.

## Conclusions

Current smokers appear to be less likely to receive cancer screening in accordance with USPSTF guidelines for colorectal, prostate, and breast cancer. Our findings suggest physicians need to be aware of this disparity, which may affect a group of individuals with a baseline higher risk for several cancer types. We recommend a concerted effort from the medical community to identify barriers to screening among smokers to implement strategies to increase acceptance and uptake of cancer screening within this population.
